# Snails associated with the coral-killing sponge *Terpios hoshinota* in Okinawa Island, Japan

**DOI:** 10.1038/s41598-021-00185-x

**Published:** 2021-10-20

**Authors:** Hideyuki Yamashiro, Hiroaki Fukumori, Siti Nurul Aini, Yurika Hirose

**Affiliations:** 1 Sesoko Station, Tropical Biosphere Research Center, University of the Ryukyus, Sesoko 3422, Motobu-cho, Okinawa 905-0227 Japan; 2 Atmosphere and Ocean Research Institute, The University of Tokyo, Kashiwa, Chiba, Japan; 3 Graduate School of Engineering and Science, University of the Ryukyus, Nishihara, Okinawa, Japan; 4 Environmental Partnership Council, Tokyo, Japan

**Keywords:** Zoology, Ecology

## Abstract

*Terpios hoshinota* is a thin encrusting sponge that overgrows live scleractinian corals and it is linked to coral loss in many reefs. However, our knowledge of the species associated with this sponge species is poor. During a periodical survey of *T. hoshinota* in 2020, we found tiny snails crawling on the sponge in the subtropical waters around Okinawa Island, Japan. We observed egg capsules inside the sponge tissue and veliger larvae released from the egg capsules. Molecular analyses of both the snails and veliger larvae (cytochrome oxidase I, COI) showed that they were identical and belonged to *Joculator* sp. (family Cerithiopsidae). There was no direct observation of predation on the sponge by this snail; however, to the best of our knowledge, this is the first report on a close association between a snail and the sponge *T. hoshinota*.

## Introduction

Coral reefs are valuable ecosystems that supply numerous services to humans, and they are home to numerous coral-associated organisms, which are linked to the high levels of biodiversity observed in these ecosystems. However, coral reefs are threatened and degraded by repeated bleaching events, owing to increasing water temperatures, ocean acidification, coral predators, infectious diseases, and physical/chemical disturbances caused by human activities^1–6^. However, sponges are predicted to be ‘winners’ in future coral reefs and, together with macroalgae, they could replace corals under a changing environment^7^. It is becoming increasingly likely that some sponges could replace corals to create sponge-dominated reefs. Changes from coral- to sponge-dominated reefs are reported in Caribbean, Atlantic, Indo-Pacific, and Pacific reefs. In Wakatobi Marine National Park, Sulawesi, Indonesia, coral coverage decreases with increasing sponges^8^. Sponge-eating organisms (spongivores) include a variety of marine species, including vertebrates such as fish and turtles; mollusks such as opisthobranchs/snails; echinoderms such as asteroids; crustaceans such as crabs, and shrimps^9,10^.

The coral-killing sponge, *Terpios hoshinota* Rützler & Muzik, 1993, is prevalent in many areas, including Guam^11,12^, Japan^13–16^, Taiwan^17,18^, the Great Barrier Reef, Australia^19^, Yongxing Island, China^20^, Malaysia^21^, Indonesia^22,23^, Maldives^24^, and Mauritius^25^.

**Figure 1:**
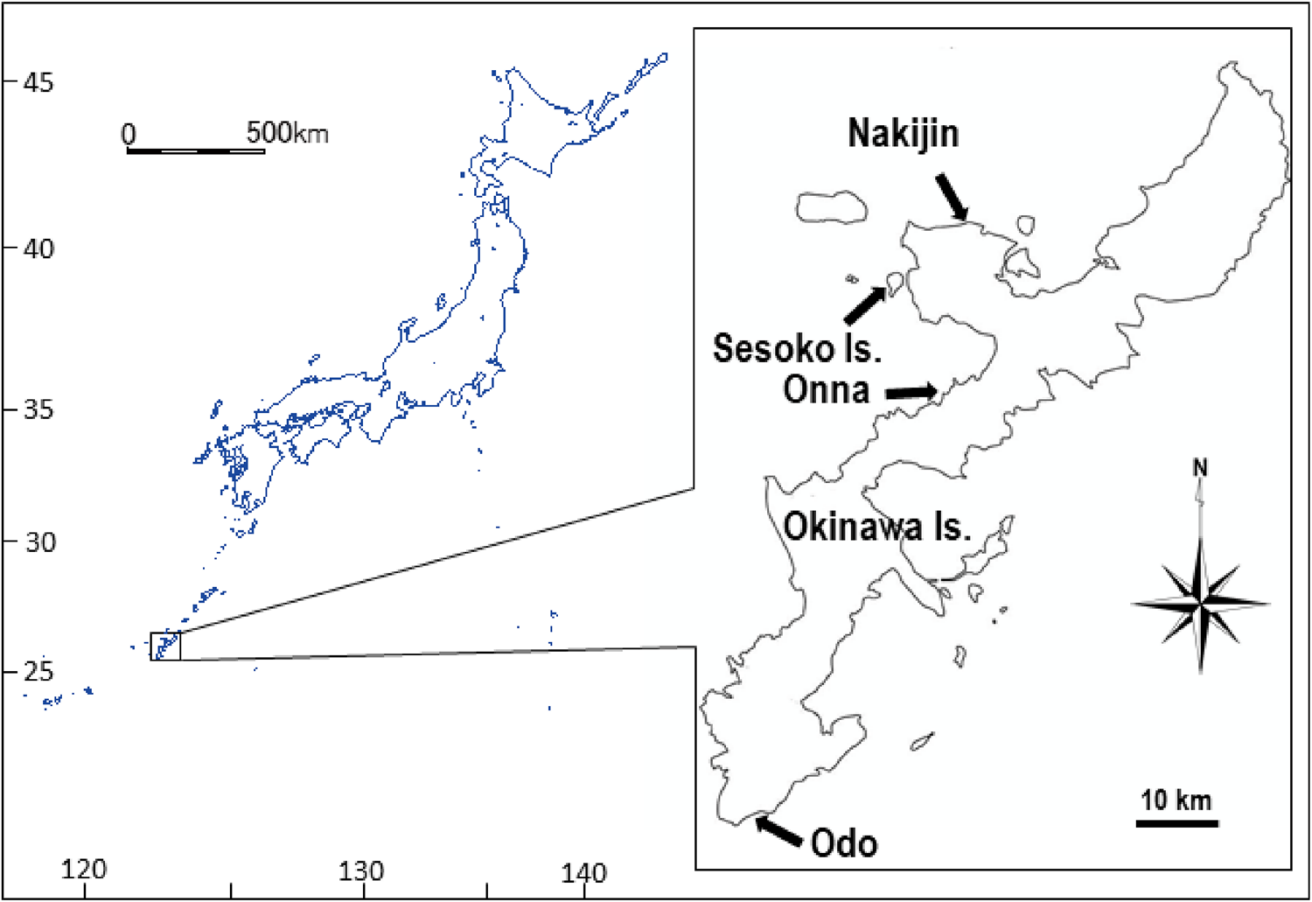
Map showing study sites. The marine station (Sesoko Station) of the University of the Ryukyus is located on Sesoko Island. The software used to create the map was HiMapMeister ver. 1.1.1, Teikoku-Shoin Co., Ltd, https://www.teikokushoin.co.jp/support/index_01.html).

**Figure 2:**
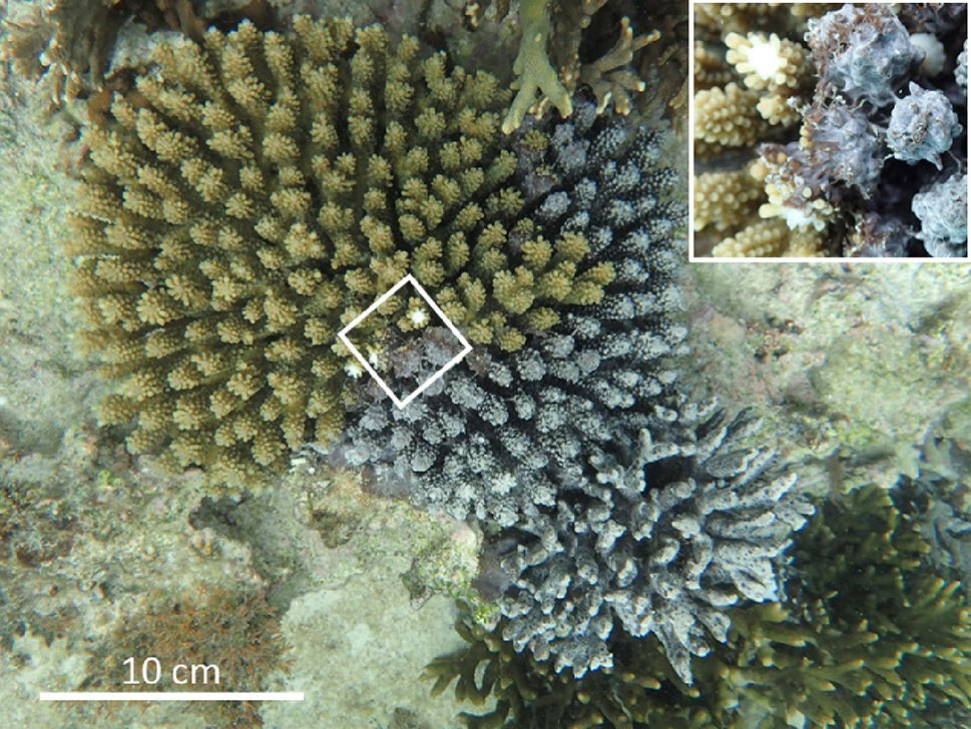
*Terpios hoshinota* covering coral colonies of *Acropora tenuis* and *Montipora digitata* on the Nakijin reef. Inset shows the sponge extending over branches of *Acropora* with thread-like tissues.

**Figure 3:**
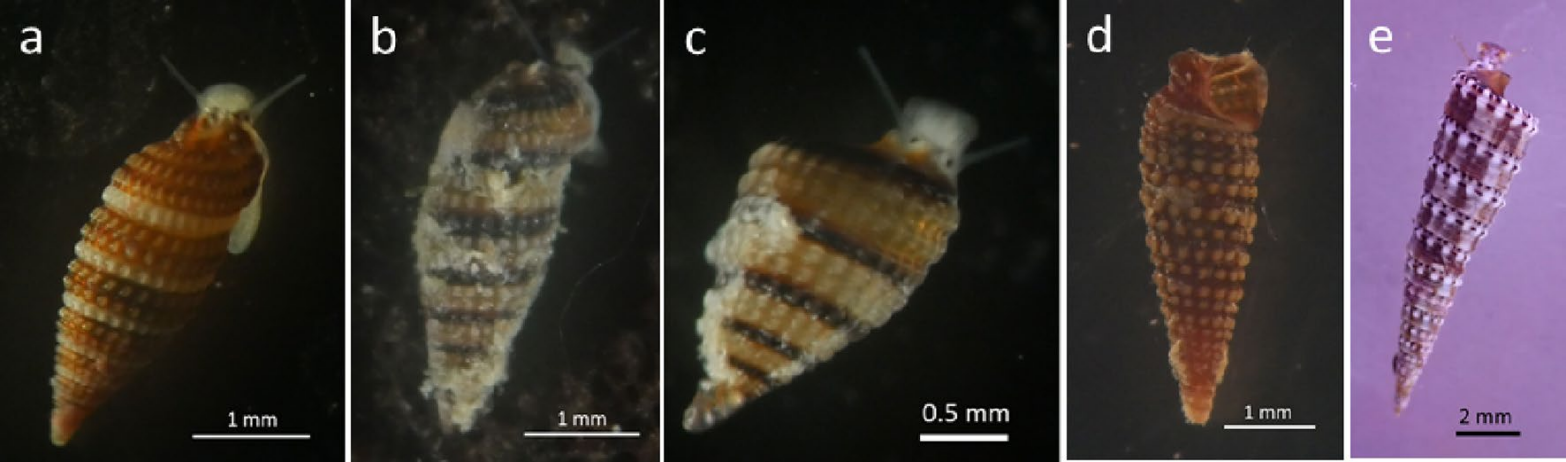
Snails collected from *Terpios hoshinota* sponge. The number of each snail collected during the study period were: (**a**) (6), (**b**,**c**) (3; **c** is a juvenile), (**d**) (1, preserved in ethanol), (**e**) (1).

**Figure 4:**
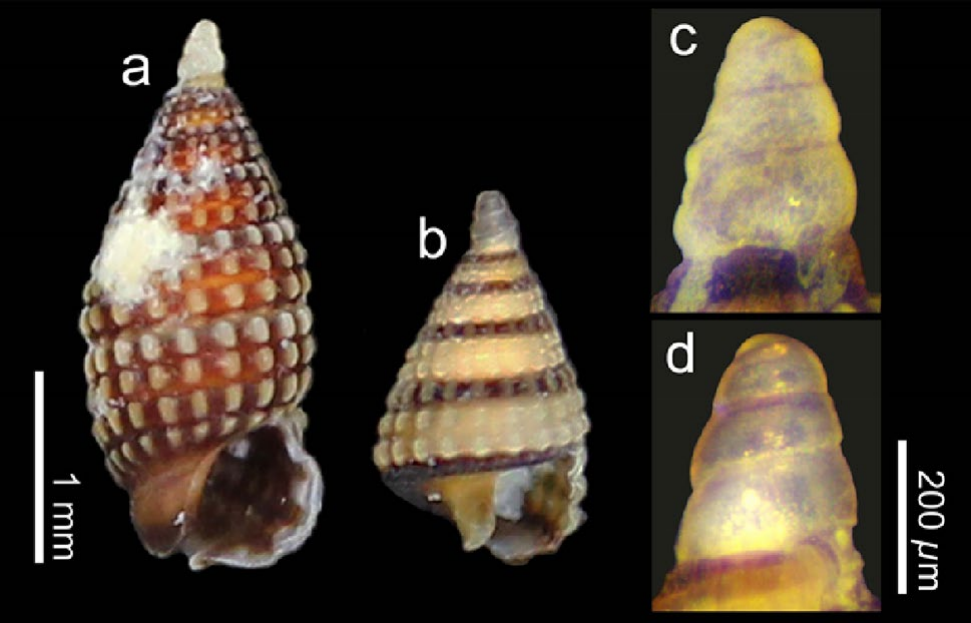
Sequenced specimens of cerithiopsid snails examined in this study. (**a**,**b**) shell. (**c**,**d**) protoconch. (**a**,**c**) *Joculator* sp. (DNA accession no. LC598716); (**b**,**d**) *Joculator* sp. (LC598717). The specimens preseved in ethanol were photographed using a microscope (LW-820T, Wraymer Inc., Japan) equipped with a digitalized camera (WRAYCAM-NOA630B, Wraymer Inc., Japan). Scale bars indicate 1 mm for (**a**) and (**b**); and 200 µm for (**c**) and (**d**).

*Terpios hoshinota* is a thin (< 1 mm thick), encrusting demosponge with numerous symbiotic cyanobacteria in its tissues. It grows rapidly on live coral at the rate of 1 mm per day (linear progression rate of 11.5–23.0 mm month^–1^) in tropical sites^11,12,25,26^. Information about its prevalence is accumulating; however, the information on the relationships between the sponge and associated species is poor. The aim of this study was to describe the snails found for the first time on *T. hoshinota*, their sites of occurrence on sponges, and to examine their relationship with *T. hoshinota*, and identify the snail using molecular DNA barcoding techniques (Figs. 1, 2, 3, 4).

## Results

**Figure 5:**
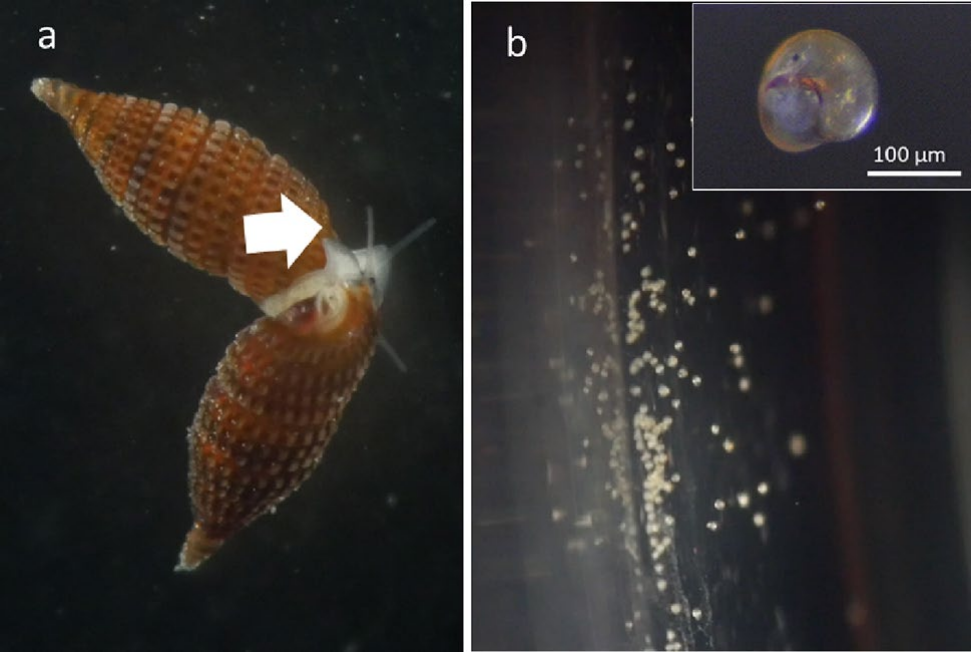
(**a**) Two snails showing mating behavior, separated from sponge, in a Petri dish. (**b**) Swimming veliger larvae toward the left (bright side) in a Petri dish. Inset shows the magnified image of a veliger larva on a slide glass.

In this study, snails crawling on the *T. hoshinota* sponge, which were overgrowing the branching coral *Montipora digitata*, were obtained from two sites around Okinawa Island (Nakijin and Odo). The material was inspected in the marine laboratory and live veliger larvae were collected from a sponge from Nakijin together with sponge larvae using a cup with nylon net (mesh size: 100 µm). Direct observation in the field and sampling were attempted in Odo, Sesoko, Nakijin, and Ogimi (> 40 branches in each site), but we failed to collect snails. This could be attributed to their small size (< 2.5 mm in shell height, Fig. 3a–c) and dark coloration. Egg capsules with veliger larvae were found in the histological sections of the specimens from Sesoko Island (January 22, July 4, 2020) and from Onna (August 4, 2020). Cerithiopsidae (Fig. 3a–c) and Triphoridae (d: *Coriophora fusca*, e: *Euthymella elegans*) snails on *T. hoshinota*e were collected from August to November 2020. The mating behavior of the two snails was observed twice in September (Suppl. Movie 1), from the snails on the Nakijin sponge’s surface and it continued even when the snails were moved to a Petri dish (Fig. 5a).

**Figure 6:**
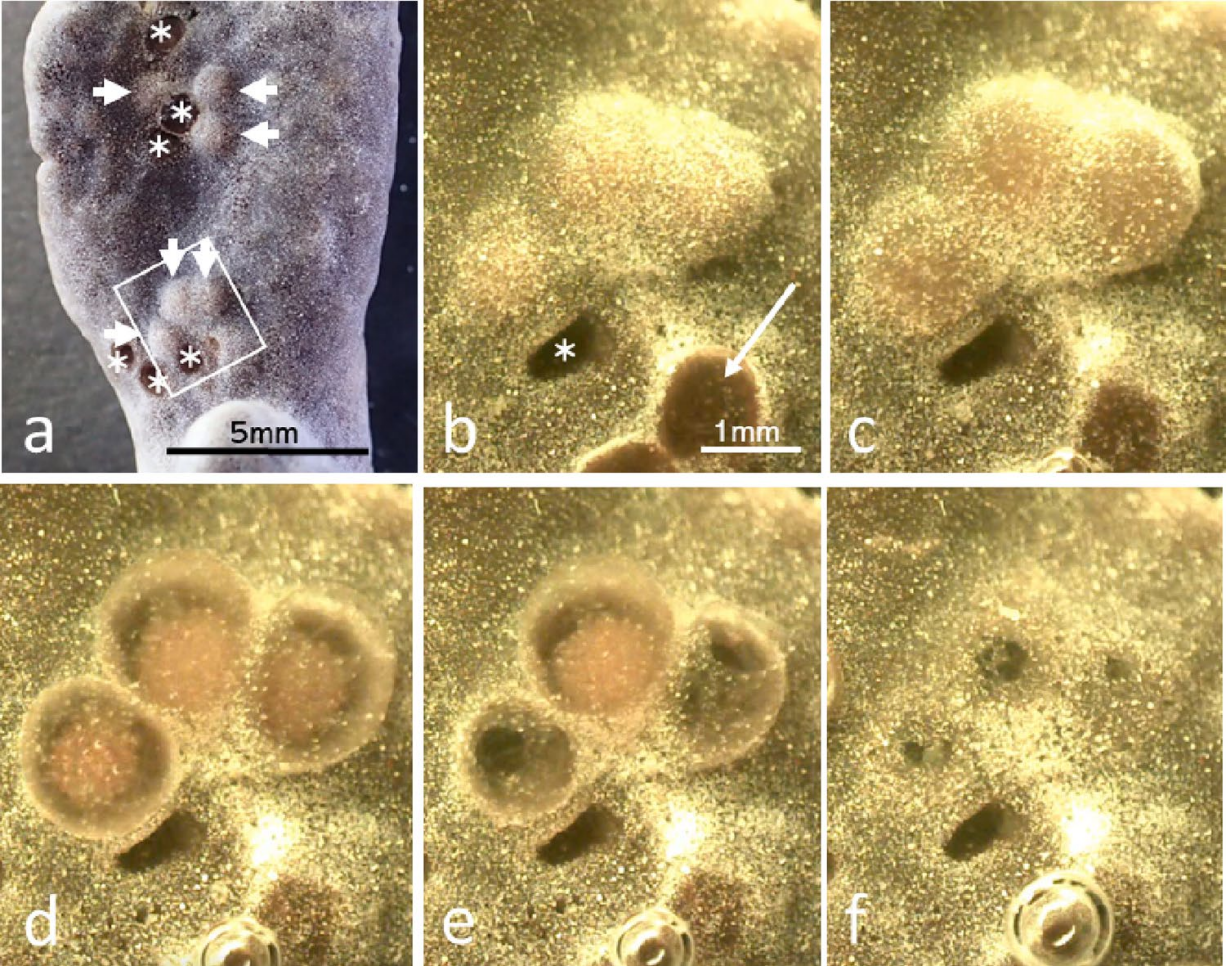
Egg capsules in the tissue of *Terpios hoshinota*. (**a**) Sponge covering the coral skeleton of *Montipora digitata*. Arrow heads: egg capsules, asterisk: holes after releasing veliger larvae. Photograph was taken with a dissecting microscope (SMZ-1000, Nikon Co., Japan). (**b**–**f**) Sequential pictures of egg capsules. Times of (**b**) to (**f**) (13:30, 17:00, 19:50, 20:00, 24:00 on December 10, 2020). The arrow in (**b**) shows the scar of a bubble released from sponge tissue. Each panel was captured from a time-lapse video using a digital microscope (Dino-Lite Premier, AnMo Elec. Co.).

Live egg capsules were found for the first time on July 24, 2020, from the Nakijin sample. Egg capsules at the stage of nearly releasing veliger larvae were visible as swollen bumps near the sponge surface (Fig. 6a, suppl. movie 2), and their size was similar to that of sponge larvae. The position of egg capsules was consistent with that of the coral calice. On the day of hatching, the egg capsules became swollen, and larvae became visible through the capsule membrane with decreasing density of sand particles trapped by the sponge. The larvae swam actively inside the capsule and then hatched, swimming out of the capsule (Fig. 6b–f). The exact time of release was observed only once in the aquarium around 8 pm on December 10 (Fig. 6e). The mean number of veliger larvae per egg capsule was 111.7 ± 17.3 (mean ± SD; range 83–132, n = 6), calculated using ethanol-fixed egg capsules. The shell length of veliger was 138.6 ± 6.0 μm (mean ± SD; range 127.3–151.5 μm, n = 51). After hatching from the egg capsule, veliger larvae started to swim and showed strong positive phototaxis toward light (Fig. 5b, suppl. movie 3). We attempted to culture the larvae in a Petri dish with filtered seawater (< 0.45 µm), but they survived only a few days.

**Figure 7:**
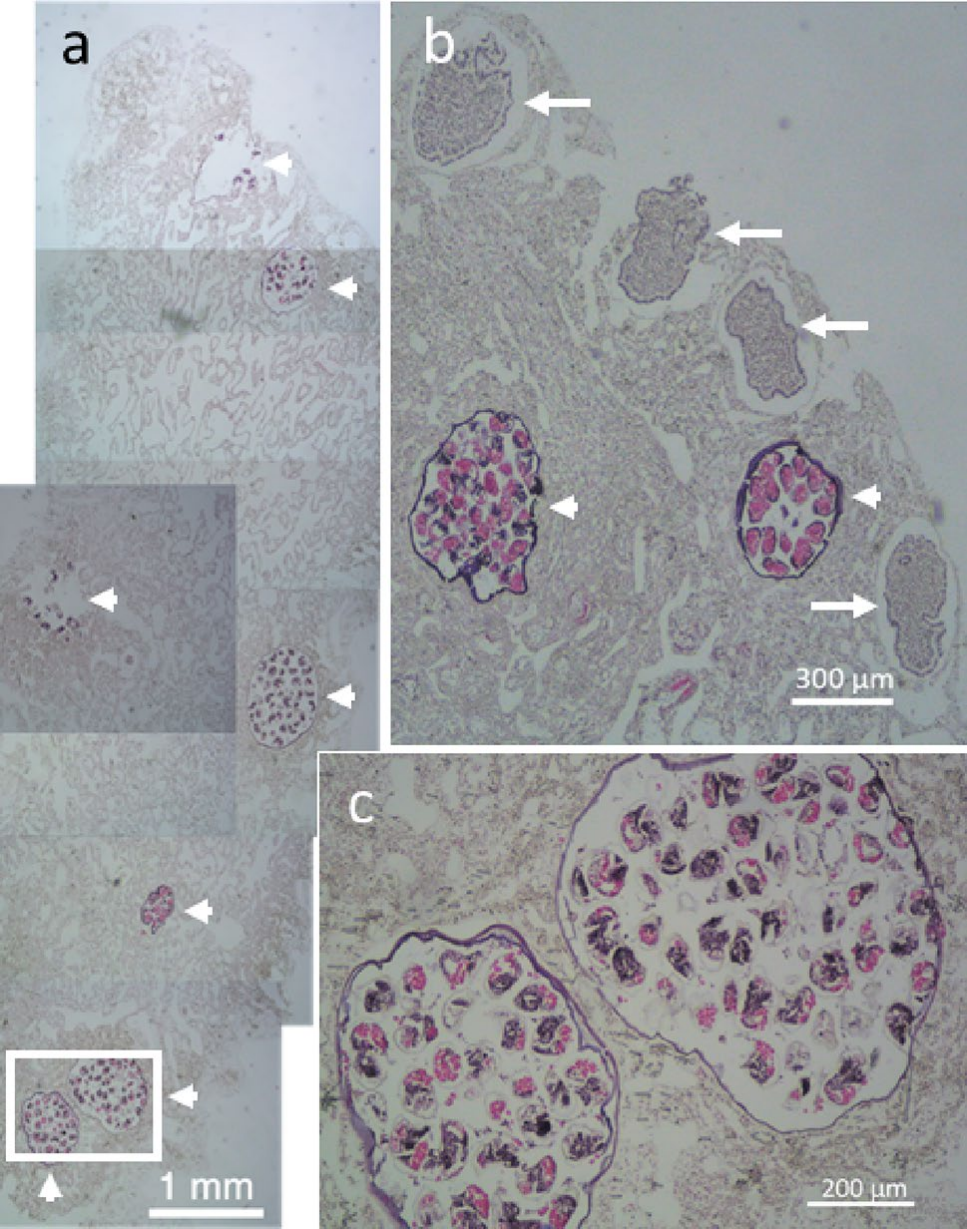
Combined histological pictures of *Terpios hoshinota* tissue. (**a**) cross section of *Montipora digitata* branch covered by *T. hoshinota* (collected from Onna, August 4, 2020). Arrow heads show egg capsules of the snail. (**b**) Sponge tissue from the specimen from Sesoko Is. (collected July 4, 2020). Arrows show sponge larvae.

Egg capsules were found in the histological sections initially prepared for observing sponge reproduction. The sponges containing egg capsules were observed in the samples obtained from Sesoko Is. on July 4 and from Onna on August 4, 2020. Figure 7 shows many egg capsules laid deep into the tissue of *Terpios hoshinota*, and the sizes of the egg capsules (1.2 mm in diameter) were close to that of sponge larvae (Fig. 7b).

**Figure 8:**
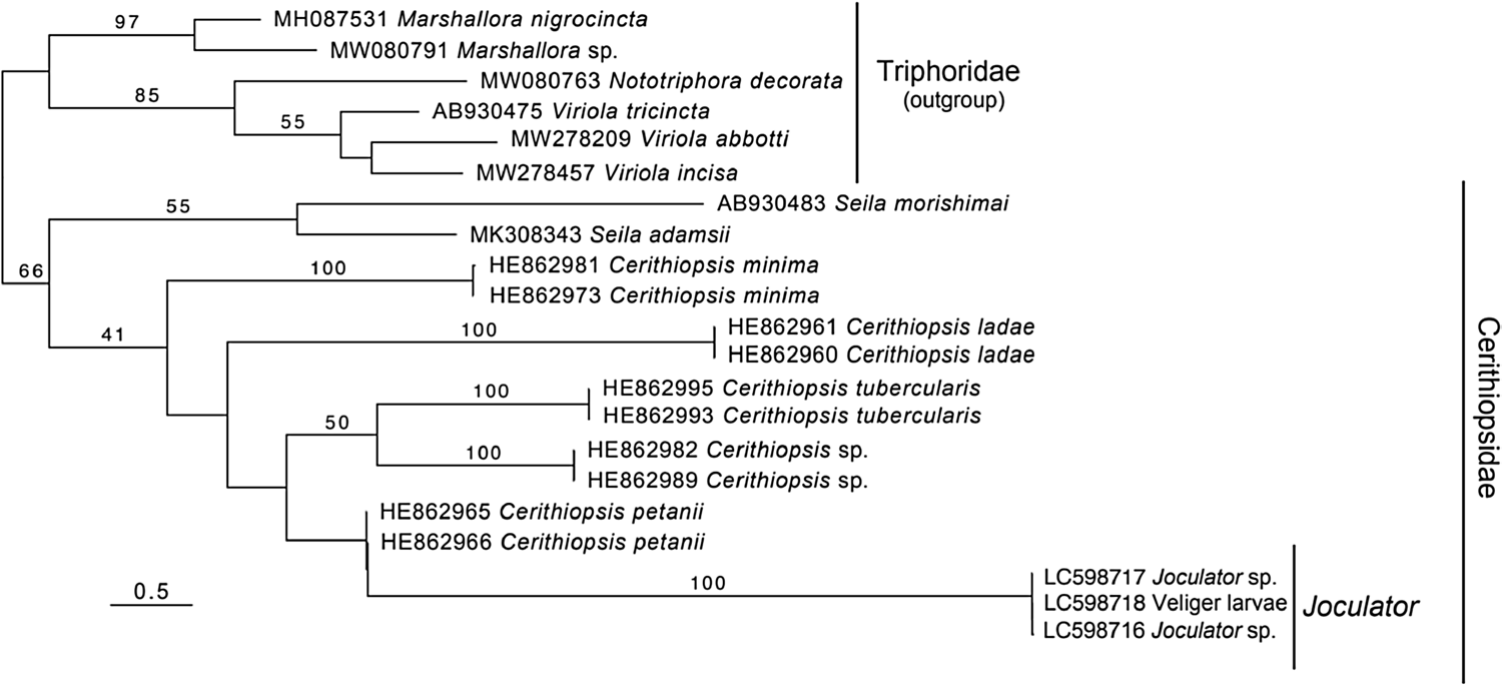
Maximum-likelihood phylogenetic tree of the family Cerithiopsidae reconstructed using the COI sequences (621 bp). Snails and veliger larvae were obtained from the sponge *Terpios hoshinota*. Bootstrap probability values for each node below 40% are not shown. Scale bar represents branch length (substitutions/site).

Molecular analyses based on COI gene sequences indicated that each snail of *a* and *b* types (Fig. 3) and the veliger larvae are the same species, *Joculator* sp. In the phylogenetic tree (Fig. 8), veliger larvae were included in a monophyletic clade with *Joculator* sp. supported by high bootstrap values (100%). In addition, low levels of genetic divergence, ranging from 0.6 to 1.5%, were observed between the two snail specimens identified as *Joculator* sp. and the veliger larvae. These values for the COI sequences of *Joculator* sp. were similar to the range of intraspecific divergences for the other cerithiopsid species (0.0 to 2.8%^40^).

## Discussion

In this study, we examined the snails associated with the sponge *Terpios hoshinota* for the first time. The number of snails observed in this study was small (< 6 individuals per species, Fig. 3); however, sponge-associated snails may be distributed widely, because snails, egg capsules, and veliger larvae were found at four Okinawa Island sites. *Terpios*-affected islands are abundant along the Ryukyu Archipelago^15^. Egg capsules and veliger larvae were observed between July and December in the present study, indicating that their reproductive season lasts for at least six months, from summer to fall.

Spongivores (sponge-eating organisms) include various animals, such as nudibranchs, snails, echinoids, fish, and turtles^9,10,26,28^. Relatively large (5–20 cm in length) dorid nudibranchs consume *Terpios* sp. in the northeastern Pacific^27,29^. *Terpios hoshinota* is a spiculate demosponge^13^ and has a cytotoxic compound^30^; therefore, this sponge is not palatable for predators. In addition, the sponge spicules (ca. 200 µm long) and particles on the surface of *T. hoshinota* tissues act as barriers to predators. However, this sponge armored with spicules, particles, and toxic substances would be a relatively safe place for snail larvae to lay their egg capsules. This study did not determine the direct evidence of the snails feeding on sponge tissues; however, there is a possibility that, like other cerithiopsids, these snails use sponges as a food source via excavation of soft tissue using their proboscises^31,32^.

In this study, we collected three different snail species from the surface of *Terpios hoshinota*. The number of snails was small; however, more intensive and quantitative surveys could find more sponge-associated snails, from the widely distributed *Terpios* in southern Japan. Therefore, survey of areas containing sponge-affected reefs along the Ryukyu Archipelago is required. It is possible that even if the sponge-associated snails consume sponges, they are unable to alter the growth of the sponge significantly, owing to their small size. Therefore, the snails are unlikely to be candidate biological control agents for inhibiting the spread of the coral-killing sponge *Terpios hoshinota*. However, studies on the species composition, geological distribution, and abundance of associates, including snails, would reveal a new view of the coral-killing sponge *Terpios hoshinota* as a host organism.

## Materials and methods

### Study sites

The study sites where the sponge-associated snails or the veliger larvae were observed include Sesoko Island (26°39′07.82″ N, 127° 51′23.26″ E), Nakijin (26°42ʹ30.9ʹʹ N, 127°56ʹ59.2ʹʹ E), Onna (26°31′39.52″ N, 127° 55′14.99″ E) and Odo (26°05′20.10″ N, 127°42′31.07″ E), all around Okinawa Island, Japan (Fig. 1). At all sites, dense aggregations of branching *Montipora* corals had developed in a shallow moat (maximum depth 2 m) together with massive *Porites* spp., foliose *M. aequituberculata*, leafy *Pavona frondifera*, corymbose *Acropora* spp., and other scleractinians. Some of these hard corals were fully or partly covered by *Terpios hoshinota* (Fig. 2). We collected the snails during the regular monthly sampling in Sesoko Island and Nakijin, during reproductive studies of the sponge, as well as from other sites where snail or veliger larvae were observed.

### Collection of snails and veliger larvae

The small size and dark color of the snails made it difficult to find them on the black sponge in the field. Most snails were found during close observation using a dissecting microscope. Veliger larvae released from the sponge were trapped in a filter cup (100 µm nylon mesh filter, cell strainer, BD Biosciences Discovery Labware) together with sponge larvae. The histological observations for the reproductive studies in sponge tissues were conducted as follows: the tissues were fixed with 10% formalin solution, dehydrated with a graded series of ethanol, embedded in paraffin, and stained with hematoxylin/eosin dyes. Presence/absence of snail egg capsules in the sponge tissue were recorded.

The snails, egg capsules, and veliger larvae were observed using a light microscope (Eclipse Ci, Nikon Co.), a dissecting microscope (SMZ-1000, Nikon Co.), and a digital microscope (Dino-Lite Premier, AnMo Elec. Co.) to obtain time-lapse images. The snails were observed in the field on the collection day; other observations and culture experiments were performed in the marine laboratory at Sesoko Station, Tropical Biosphere Research Center, University of the Ryukyus.

### Molecular identification of snails and veliger

We could collect multiple samples of only two morphological types of snails. The shell of *a* type was brown (Fig. 3a), and that of *b* type was sandy-yellow with a dark red-brown suture (Fig. 3b,c). Two cerithiopsid snails (one specimen each of *a* and *b* types; Fig. 4a,b for suture, c and d for protoconch, respectively), collected from Odo in October 2020, and approximately 60 individuals of unidentified veliger larvae were fixed and preserved in pure ethanol for morphological and molecular identification. Cerithiopsid snails were identified to the genus level based on shell morphology, as described previously^31,33–36^. In addition to the visual morphological identification, molecular identification was performed using cytochrome *c* oxidase subunit I (COI) sequences. The total DNA of snails and veliger larvae was extracted from foot tissue and 20 whole veligers, respectively, using the DNeasy Tissue Extraction Kit (Qiagen). The mitochondrial COI sequences (658 bp) were amplified through polymerase chain reaction (PCR) using the primer pairs LCO1490 and HCO2198^37^, following the conditions described earlier^38^. The PCR products were visualized through electrophoresis on a 1.5% Tris–Borate-EDTA agarose gel and purified with ExoSAP-IT (Thermo Fisher Scientific). The purified products were Sanger sequenced in both directions using an ABI 3730xl Genetic Analyzer (Applied Biosystems) at Eurofins Genomics (Tokyo, Japan). The COI sequences were manually aligned using Mesquite version 3.61^38,39^ and compared with previously reported sequences of cerithiopsid species^40,41^. Genetic divergences among the sequences were quantified using the Kimura 2-Parameter (K2P) distance model^42^ using MEGA X version 10.1.7^43^. Phylogenetic relationships of cerithiopsid species were reconstructed from COI sequences (621 bp) using the maximum-likelihood (ML) methods. The ML tree reconstruction was performed under GTR + G model in RAxML v.7.4.2^44^ with a bootstrap analysis of 1,000 pseudoreplicates. Nucleotide sequences were deposited in the DNA Data Bank of Japan (DDBJ) under the accession numbers LC598716-LC598717 for snails and LC598718 for veliger larvae. The sequenced specimens were deposited as a voucher (specimen number: 20210831-HF010-12) in the Atmosphere and Ocean Research Institute (AORI), The University of Tokyo (https://www.aori.u-tokyo.ac.jp, contact person: Hiroaki Fukumori, fukumori@aori.u-tokyo.ac.jp).

### Sampling and field studies

All necessary permits for sampling and observational field studies were obtained from the concerned authorities. Coral sampling was performed with approval from the authorities of Okinawa Prefecture, Japan.

## Supplementary Information

Supplementary Information 1.

Supplementary Video S1.

Supplementary Video S2.

Supplementary Video S3.

## Data Availability

The datasets generated or analyzed during the current study are available from the corresponding author upon reasonable request.
