# Supplementary material for: Snails associated with the coral-killing sponge Terpios hoshinota in Okinawa Island, Japan
Source: Sci Rep. 2021 Oct 20;11:20709. doi: 10.1038/s41598-021-00185-x (PMC8528886; doi:10.1038/s41598-021-00185-x)
Supplement: Supplementary file 1 — Supplementary Information 1. [file 41598_2021_185_MOESM1_ESM.pdf]

Supplementary information

Title:

Snails associated with the coral-killing sponge *Terpios hoshinota* in Okinawa Island, Japan

Authors:

Hideyuki Yamashiro, Hiroaki Fukumori, Siti Nurul Aini, Yurika Hirose

Supplementary files (MP4)

Supplement 1

Mating behavior of the snails *Joculator* sp.

Supplement 2

Time-lapse video showing the change in egg capsules and veliger larvae of the snail (December 10, 2020, taken at 500x speed).

Supplement 3

Veliger larvae gathering in the bright direction (left).
